# Preserved food for snakes: predation on naturally made “pidan” eggs in sea turtle nests on the insular beach

**DOI:** 10.1002/ecy.3477

**Published:** 2021-08-09

**Authors:** Kazumasa Matsumoto, Muneyuki Kayo, Akira Mori

**Affiliations:** ^1^ Department of Zoology Graduate School of Science Kyoto University Sakyo Kyoto 606‐8502 Japan; ^2^ Sea Turtle Association of Japan Osaka 573‐0163 Japan

**Keywords:** century egg, colubridae, foraging ecology, *Lycodon semicarinatus*, sandy beach, scavenging behavior, subtropical island

Several animal taxa store food for later consumption: for instance, squirrels and woodpeckers intentionally bury seeds in the soil and in tree trunks and consume them after a long period (Vander Wall 1990). However, exploitation of stored or preserved foods has never been reported in reptiles, although several species of snakes scavenge opportunistically on freshly dead animals such as road‐killed vertebrates (DeVault and Krochmal 2002). During a long‐term field study, we had opportunities to observe a snake (*Lycodon semicarinatus*) feeding, at least nine months after the development of embryos had stopped, on unhatched sea turtle eggs laid in a nest in the sand (Fig. 1). The unhatched eggs appeared to have escaped decomposition under special conditions, similar to the traditional preserved food, “pidan” (known as century egg). Here, we report cases that snakes utilize naturally preserved foods.

**Fig. 1:**
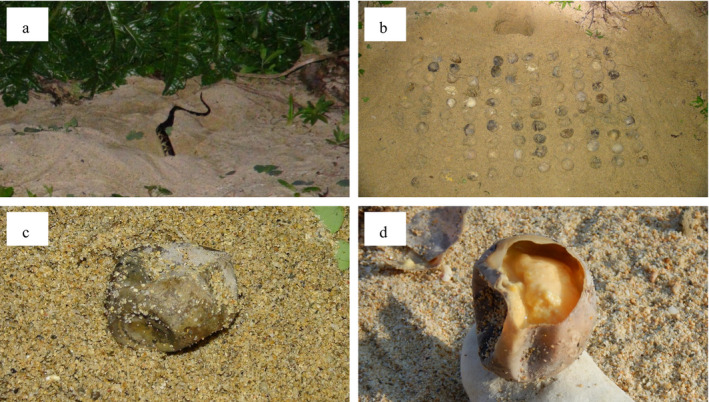
Predation by *Lycodon semicarinatus* on unhatched sea turtle eggs in the old nest. (a) A snake burrowing into the sand with only its tail exposing on the sandy beach. (b) A total of 105 unhatched sea turtle eggs excavated from the place where the snake burrowed. (c) A shrunken unhatched egg being discolored to dark brown and dark gray. (d) A semi‐solid pale yellow egg yolk of the unhatched egg.

*Lycodon semicarinatus* is a colubrid snake endemic to islands in the central region of the Ryukyu Archipelago, Japan. This snake is considered to be a nocturnal dietary generalist, preying on various small vertebrates (Hamanaka et al. 2014). A few coastal populations of *L. semicarinatus* forage on hatchlings and eggs of sea turtles (Mori et al. 1999, Matsumoto and Takaoka 2013). From April to September 2014, we conducted a field survey of the foraging behavior on the sandy beach (816 × 21 m in area; 6° in inclination from the sea shore; 26.84707° N, 128.28537° E) on Okinawa Island. Once a week, we walked on the beach every hour from around sunset to before sunrise. During the survey, we observed two occasions of unique feeding behaviors of *L*. *semicarinatus*.

The first observation was made on 15 May 2014. At 01:19, we found a male snake (snout‐to‐vent length [SVL]: 1,070 mm) burrowing into the sandy ground at a distance of 1 m from the bush line (the estimated horizontal distance between the nest and the sea shore was 19.8 m; Fig. 1a). It was obvious that the snake did not burrow into a new nest that was made by sea turtles in 2014. The snake had already inserted half of its body into the sand when we encountered it. The snake burrowed little by little until only its tail was exposed onto the surface, but the tail never entered into the sand during our observation. Seventy minutes later, the snake moved backward from the sand and completely emerged on the beach. We captured the snake immediately and force‐regurgitated its stomach content (following Carpenter 1952). The stomach contents comprised of a pale yellow paste with a small number of fine sand grains, and seemed to be the yolk of sea turtle eggs. Its wet mass was ˜15 g in total. Additional items were not found in the stomach contents.

We dug a narrow hole at the point where the snake burrowed and found 105 unhatched sea turtle eggs (Fig. 1b). We were not able to identify the species of sea turtles that the eggs belong to. The depth from the sand surface to the top of the buried clutch was ˜0.43 m (at an estimated altitude of 1.57 m above sea level), and the sand around the eggs was uniformly damp. The eggshells of nine eggs had already been torn, and a small amount of egg yolk remained inside these eggshells. The intact eggs were slightly shrunken compared to fresh sea turtle eggs (Fig. 1c). The surface of the eggshells was discolored to dark gray. By tearing the eggshell of an intact egg to examine its contents, we confirmed that the development of the embryo had stopped at the early developmental stage (Fig. 1d). The egg yolk was pale yellow and semi‐solid, which looked similar to the stomach content of the snake. Almost no egg white was identified inside the egg. All eggs were buried back into the same place after the examination.

The second observation was made on 28 May 2014. At 23:10, we found another male snake (SVL: 1,328 mm) burrowing into the same nest as the first observation. Approximately two‐thirds of its body had already been inserted into the sand. The snake continued the predation without pulling its head out from the nest during forty‐eight minutes of our observation. We captured the snake after the observation and confirmed semi‐solid yolk of old eggs in the stomach content of the snake. We released the snake without regurgitating all stomach contents to reduce stress by handling.

In 2013 and 2014, we conducted a survey on the nesting activity of sea turtles on this beach. From April to September in both years, we walked on the beach every morning to mark new nests by inserting a stick (the marking sticks were removed after hatchlings emerged from nests). From October 2013 to March 2014, during the non‐nesting season of sea turtles, we conducted the survey only once a week. The total number of nests in 2013 and 2014 was 185 (*Caretta caretta*, 177; *Chelonia mydas*, 8) and 106 (*Ca*. *caretta*, 101; *Ch*. *mydas*, 5), respectively. As the last date of sea turtle nesting in 2013 was 1 August, the old nest from where the snakes exploited the eggs had passed at least 287 d at the time of the first observation since eggs were laid. In a general view, food that has been left in nature for such a long period is not suitable for any snake to eat, unless it has undergone a biochemical transformation.

“Pidan” is an artificially preserved avian egg that is prevented from decomposition. According to the traditional recipes for pidan, salted duck eggs are made by immersing fresh eggs in a brine solution, and the eggs are covered with a mixture, containing salt, ash, and lime (calcium oxide as a strong base), and are maintained for several weeks before wrapping them with rice husks (Teng et al. 2016). The formation of pidan is processed by the penetration of alkali through the eggshell and membrane, leading to protein denaturation and hydrolysis in the egg. In general, the egg yolk and white gradually become solidified and hardened (Blunt and Wang 1918). Pidan eggs are currently classified into two categories according to whether the yolk is semi‐solid or hard‐solid. In processing eggs to obtain a semi‐solid yolk, the paste or coating fluid has less table salt and lower alkalinity than hard yolk pidan (Hou 1981). Therefore, the pH values of the egg yolk and white are relatively low (8.6 ± 0.4 [mean ± SD] and 8.7 ± 0.5, respectively; Teng et al. 2016).

The main component of sandy beaches on Okinawa Island consists of bioclastic sand such as coral fragments, shell fragments, and foraminifers (Yamanouchi 1998). The bioclastic sand is mostly constituted of calcareous substance (calcium carbonate), and therefore the soil of the bioclastic sandy beach is alkaline. In addition, the beach of the study site is occasionally covered with seawater due to typhoons and strong wind during high tide. We measured pH value at 50 cm depth on the beach by using a pH meter (SATOTECH, Kanagawa, Japan, YK‐21SP) and obtained pH values of 8.33 ± 0.12 (range = 8.18–8.58, *n* = 16). Therefore, the environmental conditions surrounding the unhatched eggs of sea turtles are similar to those that produce a semi‐solid yolk pidan.

Furthermore, the shell membrane of sea turtle eggs is relatively soft, and the calcium carbonate crystals are relatively large and sparse compared to avian eggshells (Packard and DeMarco 1991). Because of this structure, the oxygen diffusion coefficient (Ackerman and Prange 1972) and the water vapor permeability of sea turtle eggshells (Ackerman et al. 1985) are considerably larger than those of chick eggshells. These features indicate that sea turtle eggs are more easily affected by the alkaline substrate surrounding them than avian eggs. Therefore, the old unhatched eggs may have been chemically changed to a semi‐solid yolk pidan by alkali penetrating into the eggs. We considered that the non‐perishable eggs were utilized by *L*. *semicarinatus* as a preserved food in nature.

Based on the average clutch size of *Ca*. *caretta* and *Ch*. *mydas* (112.9 ± 9.3 and 108 ± 18.8, respectively; van Buskirk and Crowder 1994) and the successful hatching rate of them in other sandy beaches on Okinawa Island in 2013 (77.2% ± 18.8% and 77.3% ± 13.1%, respectively; Kawazu 2013), we estimated that more than 4,700 unhatched eggs had remained on the beach of the study site in 2013. If such a large amount of food resource available for a long period is supplied and stored each year, it can be naturally preserved foods for *L. semicarinatus*. However, many unclarified issues remain, e.g., “How much do the snakes depend on this food resource?” and “Are the changes in the eggs exactly the same as that in artificial pidan?” Continuous field surveys and chemical analysis of the eggs are necessary to confirm the ecological significance of this unique feeding system.

## Data Availability

Data (Matsumoto 2021) are available from Figshare: https://doi.org/10.6084/m9.figshare.13841192.
